# Sodium Intake Tracked from Infancy and Salt Taste Preference during Adolescence: Follow-up of a Randomized Controlled Field Trial in Brazil

**DOI:** 10.1016/j.cdnut.2022.100011

**Published:** 2022-12-22

**Authors:** Julia L. Valmorbida, Caroline N. Sangalli, Paula S. Leffa, Paola S. Baratto, Fernanda Rauber, Julie A. Mennella, Marcia R. Vitolo

**Affiliations:** 1 Nutrition Research Center, Federal University of Health Sciences of Porto Alegre, Porto Alegre, Rio Grande do Sul, Brazil; 2 Graduate Program in Pediatrics, Federal University of Health Sciences of Porto Alegre, Porto Alegre, Rio Grande do Sul, Brazil; 3 Department of Preventive Medicine, Faculty of Medicine, University of São Paulo, São Paulo, Brazil; 4 Monell Chemical Senses Center, Philadelphia, PA, USA

**Keywords:** adolescence, Brazil, child, diet, processed and ultra-processed foods, randomized controlled trial, salt taste preference, sodium

## Abstract

**Background:**

Effective interventions to promote healthy sodium intakes require understanding factors driving liking for salt taste.

**Objectives:**

To examine effects of an early feeding intervention among low-income mothers on their children’s energy and sodium intake and salt taste preferences at 12 years; and to identify age-related changes in dietary sodium sources.

**Methods:**

Secondary analyses were conducted on dietary intake and taste preference data collected from children in a longitudinal trial (NCT00629629). Mothers randomized to the intervention group received counseling on healthy eating practices for 1 year postpartum; the control group received no counseling. Two-day dietary recalls were obtained at 1 year (intervention end) and at 4-, 8-, and 12-year follow-up visits, from which foods were categorized as unprocessed, processed, or ultra-processed. At the 12-year visit, children’s most preferred concentration of salt was measured using a validated, forced-choice, paired-comparison tracking method, and pubertal stage was self-assessed.

**Results:**

The intervention group had reduced energy intake compared with controls in all food categories at 1 year (*P* = 0.04) but not at the other time points. Sodium intake from processed foods increased from 4 to 12 years and from ultra-processed foods from 1 to 4; intake from unprocessed foods decreased from 1 to 8 year (all *P* < 0.01). At 12 years, children in early stages of puberty (Tanner stages 1–3; *P* = 0.04) or in the ≥75th percentile of sodium intake (*P* < 0.01) preferred significantly higher concentrations of salt than the other children.

**Conclusions:**

Both dietary intake of sodium and early puberty were associated with preferences for higher salt concentrations. Childhood and adolescence are important periods for understanding how experience and growth shape diet by changing salt taste.

**Clinical Trial Registration:**

This manuscript reports secondary analysis of data collected in trial NCT00629629 (2001–3) and follow-up [https://clinicaltrials.gov/ct2/show/NCT00629629?term=NCT00629629&draw=2&rank=1].

## Introduction

Sodium plays a critical role in maintaining homeostasis and proper functioning of the human body [1]. However, people of all ages and from many nations are eating more sodium than is required [2, 3], due in part, to the powerful hedonic appeal of the taste of salt [4, 5], one of the main sources of dietary sodium [6]. When in excess, dietary sodium intake can be associated with increased health risks for cardiovascular diseases, especially for those deemed “salt-sensitive” [7]. Although the health conditions related to excessive sodium intake typically manifest during adulthood, their origins commonly begin in childhood [8], a period in life when salt taste preferences are heightened and when greater sodium intake is associated with heightened preferences for salty-tasting foods [5, 9].

During the past few decades, research in several countries, including Brazil, have reported excessive sodium intake among children and adolescents, with intakes rapidly increasing once complementary foods are introduced to the diet [[10], [11], [12]]. To understand the important role of early life feeding practices on setting the stage for life-long health, in 2001, we initiated a randomized controlled trial (RCT) in the city of São Leopoldo, Brazil to low-income mothers during the first year postpartum. We found that the intervention that promoted healthy feeding practices had positive effects on health and nutrition outcomes of their children; it improved breastfeeding rates at 6 and 12 mo and reduced the consumption of sugar-dense and lipid-dense foods at 1 year, and improving overall diet quality at 4 years and overall lipid profile at 8 years [[13], [14], [15], [16]].

Considering the alarming overconsumption of sodium and that the antecedents of elevated blood pressure [17], as well as food preferences [18], begin in childhood, the goal of the present secondary analysis was 2-fold. First, we evaluated whether the randomized intervention impacted sodium and energy intake at the end of the trial (1 year) and then at the 4-, 8-, and 12-year follow-up visits. We focused in particular on unprocessed, processed, and ultra-processed foods, as defined by the NOVA classification system [19], because these foods are typically high in sodium [20] and associated with unhealthy diets [21]. Second, we used a validated, forced-choice, paired-comparison method [22] to measure children’s most preferred concentration of salt at the 12-year follow-up visit. Because preference for salt is heightened during childhood [5], with the adult pattern emerging during adolescence [23], we assessed the Tanner stage of puberty for each child, hypothesizing that salt taste preferences would be heightened among those who were in the earlier stages of puberty compared with those in the culminating stages. Because dietary patterns are established early [18], childhood is an important period for understanding how the biological and experiential factors underlying salt taste sensations shape what we eat—an important influence on health in modern societies [24].

## Subjects and Methods

### Design of the RCT

The parent RCT was conducted from 2001 to 2003 in the field through home visits to low-income mothers during the first year postpartum, with follow-up visits at 4, 8, and 12 years. The RCT study design, inclusion and exclusion criteria, and CONSORT table have been published [14, 16]. The primary objective of the RCT was to determine the impact of the intervention on breastfeeding rates. We estimated a sample size of 363 dyads was needed to detect a 65% increase in the frequency of exclusive breastfeeding up to 4 mo in the intervention group (80% power, α = 0.05). All study phases were registered online at clinicaltrials.gov (NCT00629629), approved by the ethics committee of the Federal University of Health Sciences of Porto Alegre and by the Office of Regulatory Affairs of the University of Vale dos Sinos, and conducted according to the guidelines laid down in the Declaration of Helsinki. Informed consent was obtained from each mother at study entry. For each follow up-visit, we provided details on the study procedures and invited the mothers or, in rare cases, other legal caregivers (hereafter referred to as mothers) to participate. If they agreed to participate, we obtained informed consent from the mothers at each visit and also written assent from their children at the 12-year visit.

**FIGURE 1:**
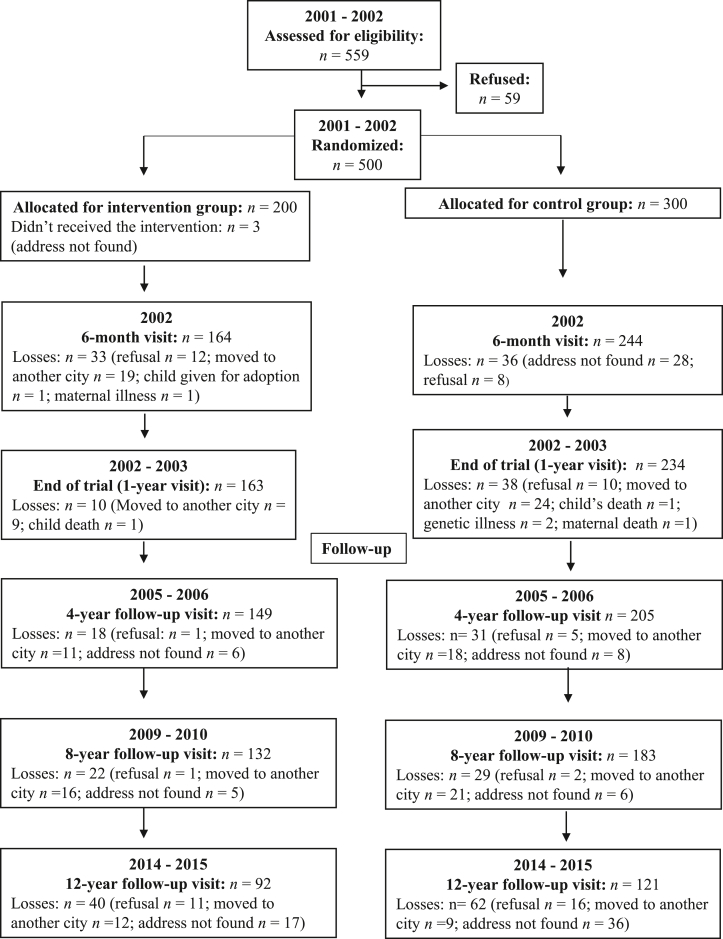
Profile of the randomized controlled trial NCT00629629 from recruitment of mother–child pairs (2001–2002) during the early postnatal period through assessment at the 12-year follow-up visit.

As shown in Figure 1, 500 mother–child pairs were randomly assigned, in a 2:3 ratio, to either the intervention or control group. For the intervention group, monthly home visits were carried out within 10 days of the child’s birth up to 6 mo, with subsequent visits at 8, 10, and 12 mo. In those visits, fieldworkers provided dietary advice based on Brazilian guidelines released the same year the study began [“Ten Steps for Healthy Eating: Feeding Guidelines for Brazilian Children from Birth to Two Years” [13]; hereafter referred to as Ten Steps]. At each home visit, counselors clarified and reinforced the dietary guidelines in accordance with the infant’s age. During the intervention period, all mothers were encouraged to maintain follow-up visits at the health care center, since the intervention did not include all health issues that arise during the first year of life. For the control group, the interviewers visited the mother–child dyads at home twice, at 6 and 12 mo, and for data collection only. No advice on infant feeding was given to the mothers, and study participation did not interfere with routine health care visits [25].

### End of trial and follow-up visits

Fieldworkers not involved in the intervention and unaware of group allocation carried out face-to-face interviews to collect data at the end of the trial and at the 4-, 8-, and 12-year home visits. Anthropometric measures and the 2-d, 24-h dietary recalls were taken in the home at the 1-, 4-, and 8-year visits, For the 12-year visit, the first day of dietary recall was conducted with the dyads in the home; the second day of dietary recall, along with psychophysical taste testing, pubertal stage evaluation, and anthropometry measurements, was conducted in the Laboratory of Gastronomy, Technology, and Innovation at the Technological Institute in Food for Health, University of Vale dos Sinos, in the presence of mothers.

### Primary outcomes

#### Dietary intake: 1, 4, 8, and 12 years

Dietary intake was assessed using 2 multiple-pass 24-h dietary recalls, which occurred on nonconsecutive days chosen randomly within a 2-wk to 1-mo period, except when children were 1 year, when only 1 dietary recall was collected. Recalls were provided by mothers when children were 1, 4, and 8 years and by the children alongside their mothers at 12 years. In the rare cases when the children spent time with a caregiver other than the mother, we interviewed the caregiver and recorded all items the children consumed during the previous day. Such 24-h recalls are regarded as highly reliable measures of dietary intake, especially when multiple days are assessed [26].

They were asked to report all the foods and beverages the child ingested the day before the interview, with guidance from trained staff. Common household measures were used to standardize portion sizes. From these dietary recalls, energy and sodium intake were estimated using Dietwin nutrition software (version 2008; Professional, Dietwin Software de Nutrição; see https://dietwin.zendesk.com/hc/pt-br). This software program is available in Portuguese and has been used by researchers to assess dietary intake in Brazilian children and adolescents [27]. The program includes 5000 food items from 3 food composition databases [[28], [29], [30]]. The amount of salt added to culinary preparations or at the table was not included in the recall because of time constraints.

We focused on dietary intake of energy and sodium from unprocessed, processed, and ultra-processed foods. When applicable, foods were categorized into either unprocessed, processed or ultra-processed food according to the NOVA classification system [19, 20], as defined in Table 1. From these data, we determined sodium (mg/d, mg/1000kcal/d) and energy (kcal/d, kcal/kg/d) intake from unprocessed and minimally processed (hereafter referred to as unprocessed), processed, and ultra-processed foods for each of the 4-time points (1, 4, 8, 12 years). To allow comparison across ages, sodium was adjusted according to energy intake (mg Na/1000kcal/d). We also identified the top 5 foods, based on intake, of each of the 3 categories and for each age (1, 4, 8, and 12 years).

**TABLE 1:** NOVA food classification system: definition for unprocessed, processed, and ultra-processed foods^1^

Food category	Definition
Unprocessed and minimally processed food	Natural foods or foods altered by processes such as drying, freezing, and pasteurization that do not add substances such as salt, sugar, oil, or fats to the original product.
Processed foods	Products made by adding sugars, oil, or salt to unprocessed or minimally processed foods, with the main purpose of increasing the durability of foods or to modify their sensory qualities (e.g., fresh unpacked breads; cheese; vegetables, fruits, and other plant foods preserved in brine or syrup).
Ultra-processed foods	Industrial formulations of substances not commonly used in culinary preparations and additives whose purpose is to imitate the sensory qualities of unprocessed or minimally processed foods or to disguise the undesirable sensory qualities of the final products (e.g., ready-to-drink milk-based beverages, ready-to-eat cakes, cookies, “instant” soups and noodles, ham, and sausages).

1 Information from Monteiro et al. [19].

#### Salt Taste Preference Assessment, 12 years

The Monell 2-series, forced-choice, and paired-comparison tracking technique for determining taste preferences [22] was first developed for measuring salt preference [31] and later modified for measuring sweet preference [32]. The method has been tested for both reliability and validity and was selected by the US National Institutes of Health Toolbox Assessment for Neurological and Behavioral Function as the method of choice to assess taste preference for clinical, epidemiological, and longitudinal studies [33].

In the present study, this method was used to determine each child’s most preferred concentration of salt in broth [22, [31], [32], [33]]. Five different taste stimuli (0.34%, 0.80%, 1.61%, 3.00%, and 5.56% wt/vol NaCl, equivalent to 0.06, 0.14, 0.28, 0.51, and 0.95 mmol/L) were made by adding varying concentrations of salt (without iodine) to a homemade NaCl-free vegetable broth made from turnips, carrots, onions, celery, black pepper, garlic, bay leaves, thyme, and parsley. Solutions were stored refrigerated for no longer than 1 wk, or frozen for no longer than 3 wk. Before testing, broths were placed in a 32°C water bath, and the temperature of each solution was verified immediately before testing.

Testing was conducted in a soundproof room with airflow control and incandescent lights that was specially designed for gastronomic and sensory taste testing. Following abstinence from eating for at least 1 h, each 12-year-old child sat opposite of 2 graduate student researchers in individual booths, who were separated by a sliding door. Before testing began, they reviewed the study procedures and answered any questions the child had, after which the sliding wood door was closed to prevent eye contact between the interviewer and the child during testing. In brief, children were presented with pairs of broth samples (30 ml each) in a disposable cup. The first pair presented was from the middle range of concentrations (0.8% and 3.0% wt/vol NaCl). Participants tasted each sample of the pair for 5 seconds and then pointed to which of the pair they liked better, without instruction on how the stimuli differed. Each subsequent pair contained the selected concentration paired with an adjacent stimulus concentration. This pattern continued until the participant chose 2 consecutive times the same concentration paired with both a higher and a lower concentration or chose the highest or lowest concentration in the series. Participants rinsed their mouth once with water after tasting each sample and twice between each pair of solutions; a 1-minute interval separated each pair presentation.

The entire task was repeated after a 3-minute break, with stimulus pairs presented in reverse order (i.e., weaker stimulus presented first in the first series, stronger stimulus first in the second series). This method controls for position bias and enables researchers to determine objectively whether the child understands the task or is responding by pointing to whatever is presented to the right or left. The geometric mean of the 2 concentrations chosen in series 1 and 2 provided the estimate of the participant’s most preferred concentration of salt.

### Secondary outcomes

#### Pubertal stage, 12 years

In a private room, with the help of a trained field worker and a photo album, each participant self-assessed their pubertal development [34, 35] and girls were asked whether they had reached menarche. We applied Tanner staging that assigns pubic hair and breast development in girls [36] and pubic hair and testicular development in boys [37]. Each adolescent was assigned a Tanner stage, which ranged from 1 (absence of pubertal signs) to 5 (final pubertal stage). If the pubic hair stage differed from mammary or testicular stage, the latter was chosen. Based on Tanner stage, children were categorized as in either early puberty (Tanner stages l–3) or late puberty (Tanner stages 4 and 5).

#### Participant characteristics

At 1 year, children were weighed without clothes and diapers, whereas on their mother’s lap, to the nearest 0.1 kg on a digital scale (Techline), and length was measured to the nearest 0.1 cm using a pediatric stadiometer (Alturexata). At 12 years, children were weighted with light clothes and no shoes to the nearest 0.1 kg on a digital scale (Techline), and standing height was measured to the nearest 0.1 cm with a stadiometer (SECA Brazil). Height-to-age and BMI-to-age Z scores were computed using WHO pediatric growth charts [38]. Through face-to-face interviews with mothers, we obtained self-reported information on their age at delivery, skin color, years of education, employment status, family income, type of delivery, and characteristics of their children, some of which were determined a priori to be covariates in the analysis because they could impact salt taste preference: birth weight, sex, maternal-reported child skin color, and BMI-to-age Z scores [5, 9, 39]. Birth weights were collected from birth records at trial enrollment.

### Statistical analysis

Analyses were conducted on de-identified data. All data were double entered by different staff members and validated in Epi Info version 6.4 (CDC, USA, see https://www.cdc.gov/epiinfo/support/downloads/previous/ei6.html). Data were analyzed using SPSS version 21.0 (IBM Statistics Inc.), and results were considered significant at *P* < 0.05. All continuous variables are presented as mean ± SEM, and categorical variables as percentages (%, *n*), unless otherwise stated. We used unpaired *t*-tests (or the Mann–Whitney *U*-tests if not normally distributed) for continuous variables and chi-square tests for categorical variables to examine differences between randomized groups at the start (enrollment), at the last visit (1 year) of the RCT and at the follow-up visits.

To determine the impact of the randomized intervention, we first assessed the relationship between energy and sodium intake at each age between the randomized groups using Spearman correlation to ensure the internal validity of the dietary methods. We then determined whether the RCT intervention had an impact on energy (Kcal/d; kcal/kg/d) and Sodium intake (mg/d; mg/1000kcal/d) from unprocessed, processed, and ultra-processed foods at 1, 4, 8, and 12 years, and the most preferred concentration of salt at 12 years. We conducted an intention-to-treat analysis [40], using data from all children regardless of study withdrawal and according to their original group randomization. We used unpaired *t*-tests (or the Mann–Whitney *U*-tests if not normally distributed) for continuous variables and chi-square tests for categorical variables. Repeated measures Friedman tests were conducted to examine the effects of time (age of child) on sodium intake, adjusted for energy (mg Na/1000kcal/d), and the food source (unprocessed, processed, ultra-processed foods). Significant effects were further examined with post hoc paired *t*-tests with Bonferroni correction.

To determine whether early nutritional intervention (randomized group), current or past dietary intake, and pubertal stage of development had interactive or independent effects on concentration of salt taste most preferred, we carried out a gradual progression from exploratory data analysis for identifying covariates to modeling, in a highly procedural manner. Significant effects were followed by splitting continuous variables into meaningful groups to illustrate findings and conduct further analyses.

First, we used bivariate analysis to examine statistically significant associations between the concentration of salt most preferred and randomized group, Tanner stage group, and dietary and sodium intake variables at 1, 4, 8, and 12 years (Mann–Whitney *U*-tests, chi-square tests, or linear regression, depending on variable characteristics). Second, we performed multivariate linear regression to explore the multiple factors associated with most preferred salt concentration at 12 years. Model 1 included those variables that were significantly correlated with most preferred salt concentration from the bivariate analyses and those identified a priori that were significantly correlated with most preferred salt concentration at 12 years. Model 2 included only the statistically significant predictors identified in Model 1, to determine if the associations remained significantly associated with salt taste preferences at 12 years. To determine if the predictors were independent or correlated, we used Pearson correlations generated in the multivariate linear regression models.

## Results

### Participants

Among the 500 mother–child dyads initially enrolled in 2001 and 2002, 79.4% were evaluated at 1 year, 70.8% at 4 years, 63.0% at 8 years, and 42.4% at 12 years (Figure 1). Of the 213 children at 12 years, 187 (80 girls, 107 boys) participated in the psychophysical taste task and were evaluated for pubertal stage.

There were no significant differences between children who were lost to follow-up and those who participated in the 12-year follow-up visit regarding randomized group assignment (*P* = 0.89), child sex (*P* = 0.73), infant delivery mode (*P* = 0.15), gestational age at birth (*P* = 0.32), birth weight (*P* = 0.81), birth length (*P* = 0.81), mother’s age (*P* = 0.24), mother’s employment (*P* = 0.40), or family income (*P* = 0.44). Except for maternal education, which was higher among those who remained in the trial (7.2 ± 2.7) than among those lost to follow-up (6.4 ± 2.7 years; *P* = 0.02), there were no differences between the randomized groups in demographic and socioeconomic characteristics at enrollment and at the 12-year visit (Table 2), neither did the groups differ in children’s height-for-age or BMI-for-age Z scores at 1, or 12 years.

**TABLE 2:** Subject characteristics at enrollment (birth), end of trial (1y) and last follow-up visit (12 y)

Characteristic	Randomized group		*P* value^1^
	Intervention	Control	
***Mothers***^2^	*n* = 176	*n* = 244	
Age at childbirth (y)	25.9 ± 0.5	25.7 ± 0.4	0.81
Education (y)^3^	6.6 ± 0.2	6.8 ± 0.2	0.41
Employment (paid)^4^	35.8% (58)	33.2% (76)	0.59
Family income < US$300^5^	75.6% (96)	67.1% (116)	0.11
Delivery by C-section^6^	35.1% (52)	41.6% (94)	0.23
***Children, at birth***^2^	*n* = 176	*n* = 244	
Sex (% girls)	44.3% (78)	44.3% (108)	0.99
Skin color (% white)^7^	40.7% (59)	45.0% (86)	0.43
Birth weight (kg)^8^	3.4 ± 0.1	3.3 ± 0.1	0.55
Birth length (cm)^8^	48.7 ± 0.1	48.8 ± 0.1	0.59
Gestational age at birth (wk)^9^	39.4 ± 0.1	39.4 ± 0.1	0.98
***Children, 1 y***	*n* = 163	*n* = 234	
Sex (% girls)	42.9% (70)	43.8% (102)	0.87
Skin color (% white)^10^	41.1% (58)	45.0% (85)	0.47
Age (mo)	12.6 ± 0.1	13.1 ± 0.1	0.42
Height-for-age Z score	–0.29 ± 0.08	–0.34 ± 0.07	0.64
BMI-for-age Z score	0.68 ± 0.08	0.58 ± 0.07	0.53
% with obesity^11^	3.1% (5)	2.1% (5)	0.56
***Children, 12 y***	*n* = 92	*n* = 121	
Sex (% girls)	42.4% (39)	39.7% (48)	0.69
Skin color (% white)^12^	39.1% (34)	45.0% (49)	0.41
Age (y)	12.4 ± 0.1	12.4 ± 0.1	0.82
Height-for-age Z score	0.19 ± 0.11	0.33 ± 0.08	0.38
BMI-for-age Z score	0.73 ± 0.14	0.62 ± 0.11	0.41
With obesity^11^	22.8% (21)	16.7% (20)	0.26

Values are % (*n*) or mean ± SEM.

1 Significant difference between groups (2-sample *t*-tests for continuous variables; chi-square tests for categorical variables).

2 Data collected at the 6-mo visit.

3 Intervention group *n* = 175, because of missing data.

4 Intervention group *n* = 162, control group *n* = 229, because of missing data.

5 Intervention group *n* = 127, control group *n* = 173, because of missing data.

6 Intervention group *n* = 148, control group *n* = 226, because of missing data.

7 Intervention group *n* = 145, control group *n* = 191, because of missing data.

8 Control group *n* = 237, because of missing data.

9 Intervention group *n* = 167, control group *n* = 230, because of missing data.

10 Intervention group *n* = 141, control group *n* = 189, because of missing data.

11 BMI-for-age Z score >3 at 1 y and >2 at 12 y [BMI Categories from the World Health Organization [27]].

12 Intervention group *n* = 87, control group *n* = 109, due to missing data.

### Dietary intake

At 1 year, children in the intervention group ate significantly less unprocessed, processed, and ultra-processed foods (kcal/d; kcal/kg/d) than those in the control group. No group differences were observed in sodium intake (mg/d; mg/1000 kcal/d) at 1, 4, 8, or 12 years (Table 3).

**TABLE 3:** Intervention effect on dietary intake of unprocessed, processed, and ultra-processed foods by randomized group assignment at 1, 4, 8, and 12 years

	Unprocessed foods			Processed foods			Ultra-processed foods		
Intake	Intervention	Control	*P* value^1^	Intervention	Control	*P* value^1^	Intervention	Control	*P* value^1^
**Age 1 y**	*n* = 154	*n* = 216		*n* = 154	*n* = 216		*n* = 154	*n* = 216	
Energy (kcal/d)	651 ± 26	724 ± 23	0.04	43 ± 511	54 ± 4	0.02	166 ± 12	205 ± 13	0.04
Energy (kcal/kg/d)	66 ± 3	73 ± 2	0.03	4 ± 1	5 ± 1	0.03	17 ± 1	21 ± 1	0.04
Sodium (mg/d)^2^	338 ± 17	374 ± 16	0.19	72 ± 8	79 ± 7	0.08	233 ± 26	300 ± 29	0.08
Sodium (mg/1000kcal/d)^2^	390 ± 17	368 ± 12	0.49	94 ± 12	84 ± 7	0.57	270 ± 31	306 ± 30	0.32
Top 5 foods	Beans			Bread^3^			Cookies^5^		
	Cow milk			Canned fruit and jam			Baby cereal^6^		
	Fresh meats			Cheese			Flavored sweetened yogurt		
	Grains and tubers			Salty canned foods^4^			Instant flavored powder drink		
	Vegetables			Died meat			Chocolate powder		
**Age 4 y**	*n* = 135	n = 187		*n* = 135	*n* = 187		*n* = 135	*n* = 187	
Energy (kcal/d)	861 ± 25	858 ± 22	0.87	110 ± 9	103 ± 7	0.42	516 ± 23	566 ± 21	0.14
Energy (kcal/kg/d)	51 ± 2	52 ± 1	0.60	7 ± 1	6 ± 1	0.67	30 ± 1	34 ± 1	0.14
Sodium (mg/d)^2^	486 ± 21	502 ± 19	0.42	175 ± 16	199 ± 16	0.76	571 ± 52	614 ± 44	0.29
Sodium (mg/1000kcal/d)^2^	326 ± 12	332 ± 11	0.93	118 ± 11	131 ± 11	0.89	375 ± 35	411 ± 32	0.29
Top 5 foods	Cow milk			Bread^3^			Sweets and desserts^7^		
	Fresh meats			Cheese			Chocolate powder		
	Grains and tubers			Canned fruit and jam			Instant flavored powder drink		
	Beans			Salty canned foods^4^			Cookies^5^		
	Vegetables			Dried meat			Carbonated soft drinks		
**Age 8 y**	*n* = 129	*n* = 176		*n* = 129	*n* = 176		*n* = 129	*n* = 176	
Energy (kcal/d)	779 ± 25	772 ± 22	0.84	110 ± 8	111 ± 7	0.77	672 ± 29	675 ± 22	0.49
Energy (kcal/kg/d)	29 ± 1	30 ± 1	0.41	4 ± 1	5 ± 1	0.48	25 ± 1	26 ± 1	0.33
Sodium (mg/d)^2^	390 ± 16	395 ± 13	0.65	546 ± 20	394 ± 16	0.16	711 ± 46	680 ± 37	0.97
Sodium (mg/1000kcal/d)^2^	256 ± 10	261 ± 8	0.78	295 ± 12	258 ± 10	0.17	443 ± 26	428 ± 19	0.89
Top 5 foods	Cow milk			Bread^3^			Sweets and desserts^7^		
	Fresh meats			Cheese			Ultra-processed meats^8^		
	Grains and tubers			Salty canned foods^4^			Cookies^5^		
	Beans			Canned fruit and jam			Carbonated soft drinks		
	Vegetables			Dried meat			Instant flavored powder drink		
**Age 12 y**	*n* = 89	*n* = 111		*n* = 89	*n* = 111		*n* = 89	*n* = 111	
Energy (kcal/d)	857 ± 38	858 ± 33	0.28	201 ± 14	236 ± 17	0.28	675 ± 41	682 ± 38	0.69
Energy (kcal/kg/d)	19 ± 1	17 ± 1	0.38	4 ± 1	5 ± 1	0.29	15 ± 1	15 ± 1	0.77
Sodium (mg/d)^2^	391 ± 19	390 ± 21	0.46	622 ± 37	675 ± 38	0.41	727 ± 49	831 ± 63	0.49
Sodium (mg/1000kcal/d)^2^	239 ± 11	237 ± 11	0.59	362 ± 17	388 ± 19	0.58	412 ± 24	474 ± 36	0.60
Top 5 foods	Fresh meats			Bread^3^			Ultra-processed meats^8^		
	Grains and tubers			Cheese			Instant flavored powder drink		
	Cow milk			Canned fruit and jam			Carbonated soft drinks		
	Beans			Salty canned foods^4^			Sweets and desserts^7^		
	Vegetables			Dried meat			Mayonnaise, margarine, and sauces		

Values are mean ± SEM. Dietary intake was assessed by the 24-h diet recall method

1 Significant difference between groups (Mann–Whitney *U*-test).

2 Total sodium does not include salt added to food during cooking or at the table.

3 Bread includes breads made with only flour, water, salt, and yeast, bought ready to eat

4 Salty canned foods include canned corn, peas, pickles, tuna, and sardines.

5 Cookies include any kind of industrially produced sweet, savory, or filled biscuit.

6 Baby cereal includes industrially produced cereal-based powder products usually added to milk.

7 Sweets and desserts include chocolate, candy, gum, ice cream, popsicles, and other sweet treats.

8 Ultra-processed meats include sausages, ham, bologna, chicken nuggets, and other processed meats.

**FIGURE 2:**
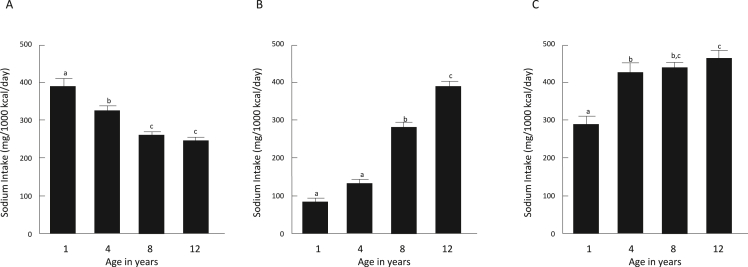
Age-related changes in sodium intake (mg/1000 kcal/d) from unprocessed foods (A), processed foods (B), and ultra-processed foods (C). Different letters represent significant differences at *P* < 0.05. Mean ± SEM, repeated measures Friedman tests; significant effects were further examined with post hoc paired *t*-tests with Bonferroni correction.

To determine how sodium intake from these foods changed over time, we focused on the 164 children for whom we had dietary data at all ages. First, we assessed the relationship between energy and sodium intake and found they were significantly correlated over time and for both groups (all *P* < 0.01), providing further evidence of internal validity of the dietary methods used. Next, we determined whether sodium intake and its sources in the children’s diet changed over time. As shown in Figure 2, daily sodium intake from unprocessed, processed, and ultra-processed foods (mg/1000kcal/d) changed with age. Sodium intake from unprocessed foods (Figure 2A**)** decreased from 1 to 4 years (*P* = 0.02) and from 4 to 8 years (*P* < 0.01) but not from 8 to 12 years (*P* = 0.80). There was no difference in sodium intake from processed foods (Figure 2B) from 1 to 4 years (*P* = 0.47) but a significant increase from 4 to 8 years (*P* < 0.01) and then from 8 to 12 years (*P* < 0.01). Sodium intake from ultra-processed foods (Figure 2C**)** increased from 1 to 4 years (*P* < 0.01) but did not change from 4 to 8 years (*P* = 0.08) or from 8 to 12 years (*P* = 1.00). The most consumed foods from each food group also differed according to age of the children at assessment (Table 3).

Because we found wide variation in sodium intake from unprocessed, processed, and ultra-processed foods at each age, for further analysis we combined sodium intake (mg/d) from unprocessed, processed, and ultra-processed foods and split children into 2 groups using the 75th percentile of intake as a cutoff point (896 mg/d at 1 year, 1611 mg/d at 4 years, 1800 mg/d at 8 years, and 2238 mg/d at 12 years), to examine effects in the highest quartile of sodium consumption.

### Pubertal stage

Of the 187 children who had their pubertal stage assessed, 62% (*n* = 116) were classified in early puberty (Tanner stages 1–3). Intervention and control groups did not significantly differ in the proportion of children in early compared with late puberty (*P* = 0.73). As expected, a higher percentage of girls than boys at 12 years were in Tanner stages 4 and 5 (*P* < 0.01). Because Tanner stage was highly associated with biological sex (*P* < 0.01), we included only Tanner stage in the subsequent multivariate analyses, to avoid multicollinearity. Sodium intake (mg/d) did not significantly differ between Tanner stages 1 and 3 (1882 ± 112 mg/d) and stages 4 and 5 (1816 ± 101; *P* = 0.12).

### Concentration of salt taste most preferred

Of the 187 children who participated in this task, 9 (5%) responded at random, 5 (3%) asked to stop before completion, and 2 (1%) were stopped by the interviewer for noncompliance with the protocol. Thus, subsequent analyses focused on the 171 adolescents for whom we have valid psychophysical data. Their most preferred concentration of salt ranged from 0.34% to 5.56% wt/vol, averaging 1.25 ± 0.08% wt/vol.

### Effects of randomized intervention group, dietary intake, and pubertal development on salt taste preferences

Although there was no significant association between randomized group and most preferred concentration of salt (*P* = 0.75), we found independent effects of pubertal development and dietary intake on salt taste preferences at 12 years. Children in Tanner stages 1–3 preferred significantly higher concentrations of salty taste (1.40 ± 0.11% wt/vol) than those in stages 4 and 5 (1.02 ± 0.10% wt/vol; *P* = 0.04). Children in the <75th percentile for sodium intake at 1 year preferred significantly higher concentrations of salt at 12 years (1.36 ± 0.10% wt/vol) than did those in the ≥75th percentile group at 1 year (0.89 ± 0.13% wt/vol; *P* = 0.01). In contrast, at 12 years, children in the ≥75th percentile preferred significantly higher concentrations of salt (1.69 ± 0.20% wt/vol) than those in the <75th percentile (1.15 ± 0.08% wt/vol; *P* < 0.01). There were no associations between sodium intake at 4 years or at 8 years and the most preferred concentration of salt at 12 years (*P* = 0.60 and 0.97, respectively).

Model 1 indicated that children in Tanner stage groups 1–3 (*P* = 0.04) or in the ≥75th percentile sodium intake group at 12 years (*P* = 0.03) preferred significantly higher concentrations of salt than those in Tanner stages 4 and 5 or in the <75th percentile, respectively. None of the covariates or sodium intake grouping at 1 year (*P* = 0.12) remained statistically associated with the most preferred concentration of salt at 12 years (Table 4).

**TABLE 4:** Output from multivariate linear regression models^1^ to identify predictors of salt taste preferences at 12 years (*n* = 162)

Predictor	β ± SEM	95% CI	*P* Value
**Model 1: *Predictors from the bivariate analysis***			
Birth weight (kg)	0.67 ± 0.23	–0.39, 0.54	0.75
Skin color (white vs. not white)	0.04 ± 0.19	–0.35, 0.43	0.83
BMI-for-age Z score at 12 y	-0.03 ± 0.08	–0.18, 0.12	0.79
Tanner stage (1–3 vs. 4-5)^2^	0.41 ± 0.19	0.03, 0.79	**0.04**
Sodium intake from unprocessed, processed, and ultra-processed foods (≥75^th^ percentile vs. <75^th^ percentile)			
At 1 y	–0.36 ± 0.23	–0.82, 0.10	0.12
At 12 y^2^	0.50 ± 0.23	0.05, 0.95	**0.03**
**Model 2: *Predictors from Model 1***			
Tanner stage (1–3 vs. 4–5)^3^	0.44 ± 0.19	0.06, 0.82	**0.02**
Sodium intake from unprocessed, processed, and ultra-processed at 12 y (≥75^th^ percentile vs. <75^th^ percentile)^3^	0.54 ± 0.21	0.13, 0.95	**0.01**

β: regression coefficient represents the mean difference (± SEM) of the most preferred concentration of salt according to variation of the independent predictor variable.

Boldface indicates significant *P* values (<0.05).

1 Model 1 included Tanner Stage, sodium intake at 1 and 12 y, which were significantly associated with salt taste preferences in the bivariate analysis and the covariates identified in the literature review; Model 2 included only those covariates that were significantly associated with salt taste preference in Model 1.

2 Pearson correlation between sodium intake groups and Tanner stage groups = –0.07, *P* = 0.21

3 Pearson correlation between sodium intake groups and Tanner stage groups = –0.01, *P* = 0.42.

**FIGURE 3:**
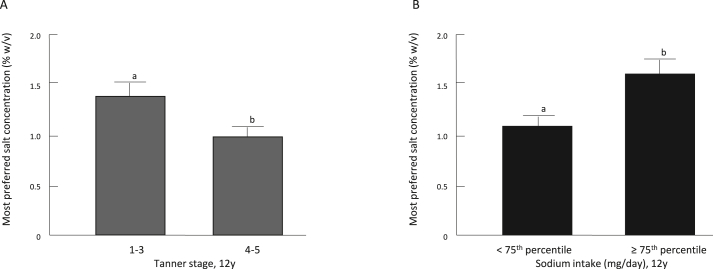
Main effects of pubertal development (A; Tanner stages 1–3 vs. 4–5; *P* = 0.04) and sodium intake from unprocessed, processed, and ultra-processed foods combined (B; <75th percentile vs. ≥75th percentile; *P* = 0.03) on most preferred salt concentration at 12 years. Mean ± SEM; multivariate linear regression, Model 2. Different letters indicate significant differences at *P* < 0.05.

Model 2 found that Tanner stage and sodium intake groupings at 12 years predicted the most preferred concentration of salt at 12 years (Table 4). Children in the early Tanner stages 1–3 preferred significantly higher salt concentrations than those in the later stages (*P* = 0.02; Figure 3A). Likewise, children in the ≥75th percentile for sodium intake at 12 years preferred higher salt concentrations than those in the <75th percentile (*P* = 0.01; Figure 3B). Tanner stage group and 12-year sodium intake group were not associated with each other as predictors of the most preferred concentration of salt (*P* = 0.42).

## Discussion

The field-based intervention, which counseled low-income mothers on exclusive breastfeeding for first 6 mo and healthy complementary feeding, had a modest yet significant effect on lowering the intake of unprocessed, processed, and ultra-processed foods, compared with the control group, when children were 1 year but not when they were 4, 8, or 12 years. Regardless of randomized group assignment, children’s energy and sodium intake from unprocessed foods decreased and their sodium intake from processed and ultra-processed foods increased, with much of these dietary changes occurring between 4 and 8 years. Modeling of these data revealed that sodium intake at 12 years and stage of puberty predicted preferences for salty taste at 12 years: the higher the dietary sodium intake and the lower the Tanner puberty stage, the higher the concentration of salt preferred. The associations of preferred salt concentration with sodium intake at 12 years and with pubertal stage were independent of each other and remained after adjustment for potential confounders.

The parent trial was originally designed to evaluate the impact of the Brazilian Ten Steps guidelines through dietary counseling among low-income mothers during the first year postpartum [13]. Positive changes, such as higher breastfeeding rates and better diet quality at the end of the trial and early follow-up visits were observed in the intervention group [[13], [14], [15], [16]], but no group effects were found for children’s sodium intake from unprocessed, processed, and ultra-processed foods. One explanation for the lack of impact of the intervention on sodium intake may be the consequence of the type of counseling received: new mothers were counseled and encouraged to focus on healthy foods in general; to avoid offering unhealthy food, such as canned goods, fried goods, soft drinks, candies, and salty snacks; and to use table salt in moderation. However, no advice was provided for processed and ultra-processed foods per se, which contain high concentrations of sodium.

An important area for future research is whether an intervention that provides guidance for mothers on how to identify high-sodium foods and educates them on the effects of food processing would be more effective in the long term than guidance on overall healthy eating. Furthermore, even for those mothers who were able to implement the guidelines, the intervention did not provide subsidies for these mothers to maintain healthy eating habits, and did not provide guidelines for how to mothers should handle challenges that arise as children grow older or for the teenagers themselves [18, 41]. What toddlers eat reflects what caregivers choose to feed them and the types of foods available in the home. In our study, regardless of the intervention effect, at 1 year children from both groups consumed mostly unprocessed foods, such as beans, milk, fresh meats, grains, and vegetables, and ultra-processed foods such as cookies without filling, baby cereals, and flavored sweetened yogurts—foods made and marketed for children [42, 43]. Mothers can influence what their children eat through purchasing and preparing food, setting rules about food and eating, acting as role models, and speaking about food with their children [44]. However, as children grow, their experiences with food extend beyond the home environment, decreasing maternal influences [41], as evidenced herein by the significant changes in children’s diets between 4 and 8 years, when unprocessed foods began to be replaced by processed and ultra-processed foods, such as sweets and desserts, cookies, and sugar-sweetened beverages.

One of the primary outcomes of the present study was the independent effects of dietary sodium and stage of puberty on salt taste preference—a primary driver for intake [45]. Dietary sodium intake and pubertal development explained the variance in salt taste preference yet were independent of each other, and significance remained after adjustment for potential confounders. Overall, the higher the sodium intake or the earlier the puberty stage at 12 years, the higher the concentration of saltiness most preferred. To our knowledge, this is the first study to measure the concentration of salt taste most preferred in Brazilian children. However, these findings are consistent with a body of research that revealed US children preferred solutions with higher salt content than did adults, with the adult-like preference patterns emerging during mid-adolescence and associated with biomarkers of growth [5, 23].

Although the causal mechanisms between dietary intake and taste preferences are not well understood, the most effective strategy known to reduce the preference for sodium-rich foods is repeated exposure to foods with little or no added salt [46, 47]. Conversely, increasing dietary salt intake increased the most preferred concentration of salt but only in individuals who had the salt added in meals; individuals who received supplemental salt in capsules showed no changes in their preferences [48]. In our study, children whose sodium intake was in the ≥75th percentile at 12 years preferred significantly higher concentrations of salt, as did those in early puberty stages. Taken together, the data suggest that a shift in preference probably relates not only to biological underpinnings of puberty but also to children’s current diet and thus current taste experiences with saltiness.

Several studies show similar dietary patterns in Brazilian children and adolescents, characterized by consumption of traditional foods, combined with a high prevalence of ultra-processed foods [12, 42, 49, 50] that are extremely attractive and relatively inexpensive and accessible [51]. These taste experiences consolidate children’s vulnerability to excess intakes as they become more independent from the family and start purchasing and eating food outside the home and being influenced by people outside the family circle, such as their peers and media, as is quite common among school-aged children and adolescents [52]. This dietary pattern results in a nutritional profile rich in sodium, as well as energy, trans and saturated fats, and free sugars [50, 53]. The addition of salt to a food reduces bitterness and increases palatability [54], which contributes in part to excessive consumption and increased the risk of cardiovascular diseases, obesity, and other diet-related diseases [55]. Thus, interventions to promote children’s healthy eating should include community-level actions, such as nutritional education in schools and food marketing regulations, to soften the impact of the food environment, full of salt-rich processed and ultra-processed foods, as observed in Brazil [50], on children’s natural preferences.

There were limitations in the present study. First, it is difficult to keep study personnel blind to the group assignment in a study involving a dietary counseling intervention [56]. However, procedures were in place such that the study personnel who collected the anthropometric and dietary intake data were not the same individuals who delivered the intervention. Further, the study personnel who administered the psychological testing were not aware of group randomization. Second, we did not measure biomarkers of growth rates, and pubertal development was self-assessed. However, a recent systematic review revealed that self-assessment of puberty is most accurate when development is categorized as into prepubertal and completing puberty phases [35], as was done in the present study. Third, lost to follow-up was high, primarily because of families moving and addresses were not forward to study; this issue has been experienced by others conducted longitudinal studies of individual living in impoverished urban areas and developing countries and remains an obstacle for research [57]. Nevertheless, we found no significant differences in baseline characteristics between those lost to follow-up and those who remained in the 12-y follow-up study, nor were there group differences in number lost to follow-up, which minimizes the possibility of bias [58, 59]. Fourth, we did not obtain information from the mothers or children on the amount of salt added during food preparation or at the table, because of time constraints and the difficulty in collecting accurate information about the salt added to all foods, which is often done without the use of cooking utensils (e.g., pinches of salt). Although the Brazilian population is gradually replacing unprocessed foods with ready-to-eat processed and ultra-processed foods [60], there is evidence that table salt remains the greatest contributor to dietary sodium intake [61]. Therefore, because of the lack of information on salt used during food preparation or at the table, the comparison of our data with other studies [2, 10, 62] is restricted. In conclusion, although there is unanimous agreement that increasing healthy dietary behaviors are the single most important aspect of reducing risks for preventable diseases [24], there is a paucity of research on how to achieve this, especially as it relates to learning to like foods lower in saltiness. The present analysis, which focused on data from children whose mothers were enrolled in an infant feeding intervention study during the first year of postnatal life, indicates that to maintain effectiveness in the long term, interventions to promote children’s healthy eating must continue to be reinforced outside as well as inside the home and combined with community-level actions, such as nutritional education in schools and food marketing regulations. In addition, our study results point to a relationship between physiological needs and the preference for saltier tastes in periods of greater growth, such as during puberty, which suggests that children and adolescents are highly vulnerable to excessive sodium consumption at this time. This, combined with the current food environment, rich in high-sodium processed and ultra-processed foods, plus the role of repeated exposures as children learn about and establish their food preferences [18], makes the development of strategies that effectively reduce sodium consumption in the long term a great challenge [6, 47]. Thus, developing evidence-based strategies that promote healthy eating during the vulnerable periods of childhood and adolescence, when preferences are set, should be a public health investment priority.

## Funding

Supported by the Ministry of Health - Brasilia, Distrito Federal; Brazil (no. 577/200), and Fundação de Amparo à Pesquisa do Rio Grande do Sul – Porto Alegre, Rio Grande do Sul; Brazil (PPSUS/2006/1537-7) and by the National Council for Scientific and Technological Development–CNPq – Brasília, Distrito Federal; Brazil (grant 476119/2008-1), conceded to MRV. JAM was supported by grant R01DC016616 from the National Institute of Deafness and Other Communication Disorders, Bethesda, MD, USA. None of the entities providing support were involved in the design, implementation, analysis, interpretation of the data, or in the production of the final manuscript.

## Author disclosures

The authors report no conflicts of interest.

## Data Availability

Data described in the article, code book, and analytic code will be made available upon request pending adequate permissions.

## Acknowledgments

We thank the families who participated in the study.

## Author contribution

The authors’ responsibilities were as follows – JLV co-drafted and revised the manuscript and carried out statistical analysis. CNS, PSL, and FR supervised data collection and revised the manuscript. PSB revised the manuscript. JAM supervised statistical analyses and co-drafted and revised the manuscript. MRV conceptualized and designed the trial, coordinated and supervised data collection, storage, and de-identification; co-drafted and revised the manuscript and had primary responsibility for final content. All authors approved the final manuscript as submitted and agree to be accountable for all aspects of the work.
